# The persisting influence of organized sports participation on mental health and wellness: a longitudinal repeated measures study of adolescent female athletes

**DOI:** 10.3389/fspor.2025.1527622

**Published:** 2025-04-28

**Authors:** Rachel Meyers, Madison L. Brna, Veronica Hogg-Cornejo, Catherine Donahue, Emily A. Sweeney, Aubrey Armento, David R. Howell

**Affiliations:** ^1^ Sports Medicine Center, Children’s Hospital Colorado, Aurora, CO, United States; ^2^ Department of Orthopedics, University of Colorado School of Medicine, Aurora, CO, United States; ^3^ University of North Carolina School of Medicine, Chapel Hill, NC, United States

**Keywords:** female adolescent athletes, mental health, sports participation, physical activity, athlete

## Abstract

**Purpose:**

We examined if female adolescent athletes demonstrated changes on mental health measures 6-months after the end of a sport season compared to an immediate post-season assessment, and whether those who were actively playing sports 6-months after the end of the season reported different mental health ratings compared to athletes who were not.

**Methods:**

Participants underwent three assessments: a pre-season, immediate post-season, and final 6-month post-season assessment on measures of anxiety ratings, depressive symptoms, grit, peer relationships, fatigue, and body appreciation.

**Results:**

Participants reported significantly higher anxiety ratings at 6 months post-season compared to immediately post-season (6.6 ± 4.2 vs. 7.7 ± 4.5; *p* = 0.02), but no significant differences across time for other outcome measures. Those who were actively participating in sports at the 6-month follow-up reported significantly higher body appreciation ratings than those who were not (41.7 ± 5.9 vs. 37.1 ± 7.6; *p* = 0.01).

**Discussion:**

Our findings indicate active sports participation is associated with higher body appreciation among female athletes.

## Introduction

Roughly 27 million individuals between the ages of 6–17 years participate in sports in the United States (1). Sports participation provides many benefits to both physical and psychological health (2–4), including improved mood, decreased risk for depressive and anxiety symptoms, improved social relationships, and overall quality of life (5, 6). These benefits may be particularly pronounced during adolescence, as physically active youth have less severe depressive symptoms compared to youth who do not participate in sports (7). However, less is known on the short and long-term psychological effects of sports participation among adolescent athletes. As such, it is imperative to understand the short and long-term associations between sports participation and psychological health in the adolescent population, including anxiety ratings, depressive symptoms, grit, fatigue, peer relationships, and body appreciation.

Participating in sports during adolescence has been found to improve anxiety and depressive symptoms later in life (8–11). Adolescent recreational sport athletes with at least 2 years of participation had lower levels of anxiety and depressive symptoms compared to participants that did not participate in recreational sports (8). Furthermore, adolescents participating in organized sports for more than 1 year had better mental health outcomes (i.e., less anxiety and depressive symptoms) compared to those who did not participate in organized sports, with the strongest association being among those who participated in organized sports for 4–5 years (8). Additionally, involvement in school sports during adolescence has been associated with less severe depressive symptoms in young adulthood (9). However, adolescent female athletes reported more severe anxiety and depressive symptoms than their male peers, and consequently, had higher sports participation dropout rates (12, 13). Thus, it is critical to identify how mental health and wellness measures change over the course of time during adolescence, particularly among female adolescent athletes.

Although studies have examined the association of sports participation and anxiety and depressive symptoms later in life, it is not well understood how sports participation may be influenced by one's grit. Grit is defined as the persistance to strive for long-term goals despite setbacks and challenges (14, 15). Tedesqui and Bradley found that grit scores were not associated with previous sports participation (16), while Cocic et al. demonstrated that organized sports participation among elite youth soccer players was assoicated with higher grit levels (17). Higher grit levels were also found to be associated with less anxiety and depressive symptoms among non-athletes (15). However, to the best of our knowledge, no studies to date have examined how one's grit may change from a post-season assessment to a longer-term (e.g., 6-month) follow-up assessment among female adolescents. Thus, it is important to better understand the longitudinal changes of grit and sports participation among this population.

In addition to anxiety symptoms, depressive symptoms, and grit, other psychological components such as peer relationships, fatigue, and body image may also each be modified through organized sports participation (18–21). Among school-aged children, researchers identified a positive association between peer relationships and sports participation (18). Furthermore, persistent physical inactivity in adolescents (exercise <3 times/month) is associated with poor long-term (over 1–2 years) peer relationships (19). Lastly, youth who participated in both individual and team sports or team sports alone had greater peer relationships compared with children who just participated in individual sports (20). Participating in organized sports has also been found to reduce fatigue and improve body image among adolescent females compared to those who were not actively involved in sports (21). Of note, psychological benefits were strongest for girls who participated in sports compared to boys, and differed based on sport type (21). However, most of these findings were in both male and female adolescents, and little remains known on the longitudinal effects of sports participation and peer relationships, fatigue, and body image among female adolescent athletes, specifically. Therefore, understanding how sports participation affects psychological measures at various points in time, such as immediately post-season and 6 months after the end of the season, may give better insight into promoting female adolescent health outcomes.

The primary purpose of this study was to examine if female adolescent athletes demonstrated changes on measures of mental health and wellness (anxiety ratings, depressive symptoms, grit, fatigue, peer relationships, and body appreciation) 6-months following the end of a sport season compared to an immediate post-season assessment. We hypothesized that mental health measures for each domain (anxiety ratings, depressive symptoms, grit, fatigue, peer relationships, and body appreciation) would be worse 6 months after the end of the season compared to the post-season assessment. Our secondary purpose was then to examine if adolescent athletes who were actively participating in organized sports 6 months after the end of the season reported different mental health and wellness ratings compared to athletes who were no longer participating in organized sports at the 6-month follow-up.

## Methods

### Study participants and study design

We conducted a longitudinal repeated measures study of female adolescents who were participating in a season of flag football throughout the Denver Metro Area in the fall/spring of 2023/2024. Potential participants were included in the study if they were active participants of a flag football team at a local high school and had no injury that limited physical activity participation at the time of enrollment. Study participants were recruited by high school coaches and local community networks through distribution of study flyers. Participants enrolled and underwent an initial pre-season assessment in summer 2023, completed an immediate post-season assessment in fall 2023, and returned in spring 2024 for a final 6-month post-season follow-up assement. At all time points, participants completed assessments detailing demographics, medical history, recent physical activity, and the primary outcome variables (anxiety ratings, depressive symptoms, grit, fatigue, peer relationships, and body appreciation). Immediate post-season and 6 months post-season assessments constituted the primary time points for analysis. The study protocol was approved by the Colorado Multiple Institutional Review Board before study commencement and all participants and their parents (if under 18 years of age) completed consent/assent prior to completing any study procedures.

### Outcome measures

Participants completed questionnaires that evaluated six domains: anxiety ratings, depressive symptoms, grit, peer relationships, fatigue, and body appreciation. The *Hospital Anxiety and Depression Scale* (HADS) was used to assess for severity of anxiety and depressive symptoms, which has previously been validated in adolescent athletes (22), and has been observed as a reliable scale (23). Participants were instructed to answer 7 questions, each ranked on a 4-point Likert scale with higher numbers representing more severe/frequent symptoms. Scores were calculated as the sum of all responses ranging from 0–21. Scores within the ranges of 0–7 were classified as normal (no depression or anxiety), scores within the ranges of 8–10 were classified as borderline abnormal (suggestive of minor anxiety/depression), and within the ranges of 11–15 were classified as moderate anxiety/depression, and scores ≥16 were classified as severe anxiety/depression (22).

The *Short Grit Scale,* previously validated for adolescents*,* was used to assess for grit scores (24). Participants answered 8 questions, each ranked on a 5-point Likert scale. Participants could answer options that ranged from “very much like me” to “not like me at all.” A few examples of the questions asked include (1) setbacks don't discourage me, (2) I finish whatever I begin, or (3) I am diligent. Scores were calculated as the sum of all responses divided by the number of questions answered with the maximum score being 5 (extremely gritty) and the lowest score being 1 (not at all gritty). This scale has been established as a reliable measure among adolescents (25).

The *Patient-Reported Outcomes Measurement Information System (PROMIS)*, Pediatric Global 37 v. 1.1 peer relationship domain was used to assess peer relationships, which has also been validated in adolescents. Participants ranked 6 questions on a 5-point Likert scale, reflecting the frequency of perceptions around peer relationships. The total sum of answers created “raw scores” which were converted to “T-scores”, with a higher score indicating stronger peer relationships and support. Among uninjured adolescents, 50 is the average rating with a score between 45 and 55 being considered “normal.” A score below 45 is considered a below average rating (little to no support from peers), while a score above 55 is considered an above average rating (26–28). This tool has established acceptable test-retest reliability among adolescents (29).

To assess for fatigue, participants answered questions from the PROMIS Global 37 v. 1.1 Fatigue-domain. It involved 6 questions, each ranked on a 6-point Likert scale (reflecting the reason behind fatigue). Participants could choose the answer options: (1) being tired made it hard for me to keep up with my school work, (2) I got tired easily, (3) I was too tired to do sports or exercise, (4) I was too tired to enjoy the things I like to do, (5) I felt weak, or (6) I had trouble finishing things because I was too tired. The scoring system was the same as described above for peer relationships. After T-score conversion, 50 is the average rating, a score under 45 is less fatigue than average, and greater than 55 is more fatigue than average (30–32).

The *Body Appreciation Scale,* previously validated in both male and female adolescents*,* was utilized to assess for body image ratings among female adolescents. Participants answered 10 questions about individuals' acceptance of, favorable opinions towards, and respect for their bodies. Each question is rated on a scale of 1 (Never) to 5 (Always), resulting in a global score ranging from 5–50 with higher scores indicating a higher body image perception (33, 34). It has been established as a psychometrically sound and clinically useful measure of body appreciation (34).

### Grouping variable: active organized sports participation at 6-month follow-up

At the 6-month assessment, we asked participants whether they were actively participating in sports. Specifically, we assessed whether they had participated in one or more organized sport over the past month, and to identify which specific organized sports (practices and/or games, structured time with a coach present) they had participated in. We then grouped participants into those who did and did not report active sport participation at this 6-month follow-up time point. Furthermore, we also asked participants to report the total number of hours of physical activity engagement in the past week to provide an estimate of overall physical activity participation at this 6-month follow-up assessment. Specifically, participants were asked to report the average number of minutes of physical activity they performed in the week prior to the assessment.

### Childhood opportunity index rating

To provide understanding of the generalizability of our findings, we calculated two zip code measures (child opportunity levels and child opportunity scores) to understand the childhood opportunity index (COI) rating. COI scores can be used to compare neighborhood opportunity on a scale from 1 (lowest neighborhood conditions) to 100 (highest neighborhood conditions) (35). All neighborhoods are ranked nationally in terms of their overall COI z-scores and get divided into 100 rank-ordered groups. Each of these groups contain 1% of the child population and is assigned a child opportunity score from 1–100. (35).

### Statistical analysis

Descriptive statistics are presented as mean (standard deviation) for continuous variables, median (interquartile range) for count variables, and number of participants within group (corresponding percentage) for categorical variables. For our primary purpose, we compared each of the six rating outcome measurements between the immediate post-season and 6-month post-season follow-up time points using paired samples t-tests.

For our secondary purpose, we compared each of the six rating outcome measurements between those who did and did not report active organized sport participation at the 6-month follow-up assessment using independent t-tests. We also calculated Cohen's d effect sizes to interpret clinical meaningfulness for these between group comparisons, defined as *d* > 0.2 = small effect, *d* > 0.5 = medium effect, *d* > 0.8 = large effect (36). We then constructed a series of linear regression models to adjust for covariates. In each model, the outcome was the 6-month outcome measure (e.g., HADS-anxiety), the predictor was whether the participant reported active organized sports participation at the 6-month follow-up assessment (yes/no), and covariates were the post-season outcome measure (e.g., HADS-anxiety post-season), total physical activity hours/week at the 6-month assessment, age, and BMI. We did not perform a *post-hoc* power analysis, as this may suggest that the any absence of significance in our study would be purely due to sample size limitations, without considering the possibility that the observed effect may genuinely lack practical or clinical relevance (37). Statistical significance was defined as *p* < 0.05. All statistical analyses were two-sided and preformed using Stata Statistical Software: Version 18 (StataCorp, LLC, College Station, TX, USA).

## Results

We enrolled 84 participants at the start of the athletic season, all who identified that they would be participating in flag football in the upcoming season. Of the 84 participants who were initially enrolled in the study, 13 did not return for the immediate post-season follow-up, and 12 did not return for the 6-month post-season follow-up. As such, those 25 individuals were excluded from the analysis, and our study included a total of 59 female adolescent athletes. Those who were included and excluded from analysis were not significantly different in their age at the time of enrollment (16.3 ± 1.1 vs. 16.6 ± 1.0 years of age; *p* = 0.21). All participants indicated that they lived in zip codes in the top 50th percentile of socioeconomic advantage nationally (Table 1). While many participants reported flag football was the primary sport that they participated in, other sports were also identified (e.g., soccer, basketball, lacrosse) as primary sports (i.e., flag football was a secondary sport) (Table 1).

**Table 1:** Descriptive characteristics of the participants who enrolled in the study and completed the post-season and 6-month follow up evaluations (*N* = 59).

Continuous variables		Mean (SD)	Range
Age (years)		16.3 (1.1)	14.2–18.2
Time between visits (days)		172.6 (6.9)	151–185
Height (cm)		165.2 (8.3)	145–191
Weight (kg)		60.0 (9.9)	45–93
BMI (kg/m^2^)		21.9 (2.5)	17.3–29.8
Area deprivation index national percentile rank		18.1 (9.3)	5–45
Childhood opportunity index rating		84.3 (15.9)	23–100
Categorical variables		*N* (% within sample)	
Gender	Girl	57 (97%)	
	Non-binary	2 (3%)	
Race	American Indian or Alaska Native	1 (2%)	
	Asian	4 (7%)	
	Black or African American	4 (7%)	
	White	42 (71%)	
	Native Hawaiian or Pacific Islander	2 (3%)	
	More than one race	6 (10%)	
Ethnicity (Hispanic or Latina/x)		10 (17%)	
History of anxiety		13 (22%)	
History of depression		7 (12%)	
Primary sport	Flag football	25 (42%)	
	Soccer	10 (29%)	
	Basketball	6 (17%)	
	Lacrosse	4 (11%)	
	Track & Field- Track Events	3 (9%)	
	Volleyball	3 (9%)	
	Golf	2 (6%)	
	Diving	1 (3%)	
	Gymnastics	1 (3%)	
	Rugby	1 (3%)	
	Swimming	1 (3%)	
	Tennis	1 (3%)	
	Wrestling	1 (3%)	

**Figure 1:**
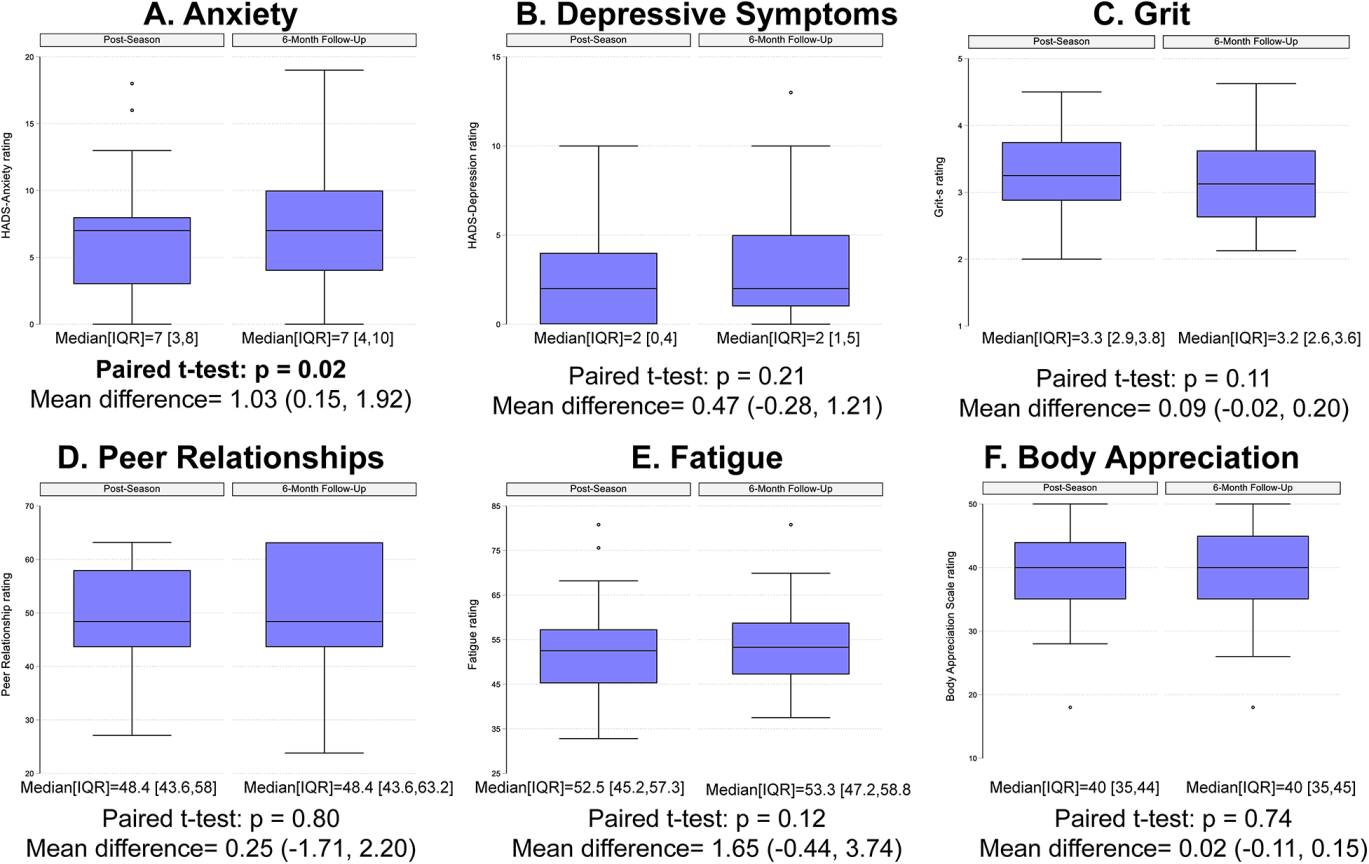
Box plots describing the self-reported ratings for **(A)** anxiety, **(B)** depressive symptoms, **(C)** grit, **(D)** peer relationships, **(E)** fatigue, and **(F)** body appreciation at the post-season and 6-month post-season follow-up time points.

For our primary purpose, we observed that participants reported significantly higher anxiety ratings at the 6-month follow-up assessment compared to the immediate post-season assessment (median [interquartile range] at post-season = 7 [(3, 8)] vs. 6-month follow-up = 7 [(4, 10)]; Figure 1A). However, there were no significant differences between time points for depressive symptoms (Figure 1B), grit (Figure 1C), peer relationships (Figure 1D), fatigue (Figure 1E), or body appreciation (Figure 1F).

For our secondary purpose, we observed that *N* = 32 (54%) of the enrolled participants reported active organized sport participation at the 6-month follow-up assessment. Those who reported active sports participation also reported significantly higher volumes of physical activity overall per week than those who did not (10.5 ± 7.2 h/week vs. 4.4 ± 6.0 h/week; *p* < 0.001; mean difference = 6.14, 95% CI = 3.01, 9.27). Among those who reported active participation in sports, the median number of sports played was 1 [IQR = 0, 1]. In contrast, the two groups did not significantly differ in their physical activity volume at the initial pre-season evaluation (8.1 ± 6.5 h/week vs. 7.2 ± 3.8 h/week; *p* = 0.53).

**Figure 2:**
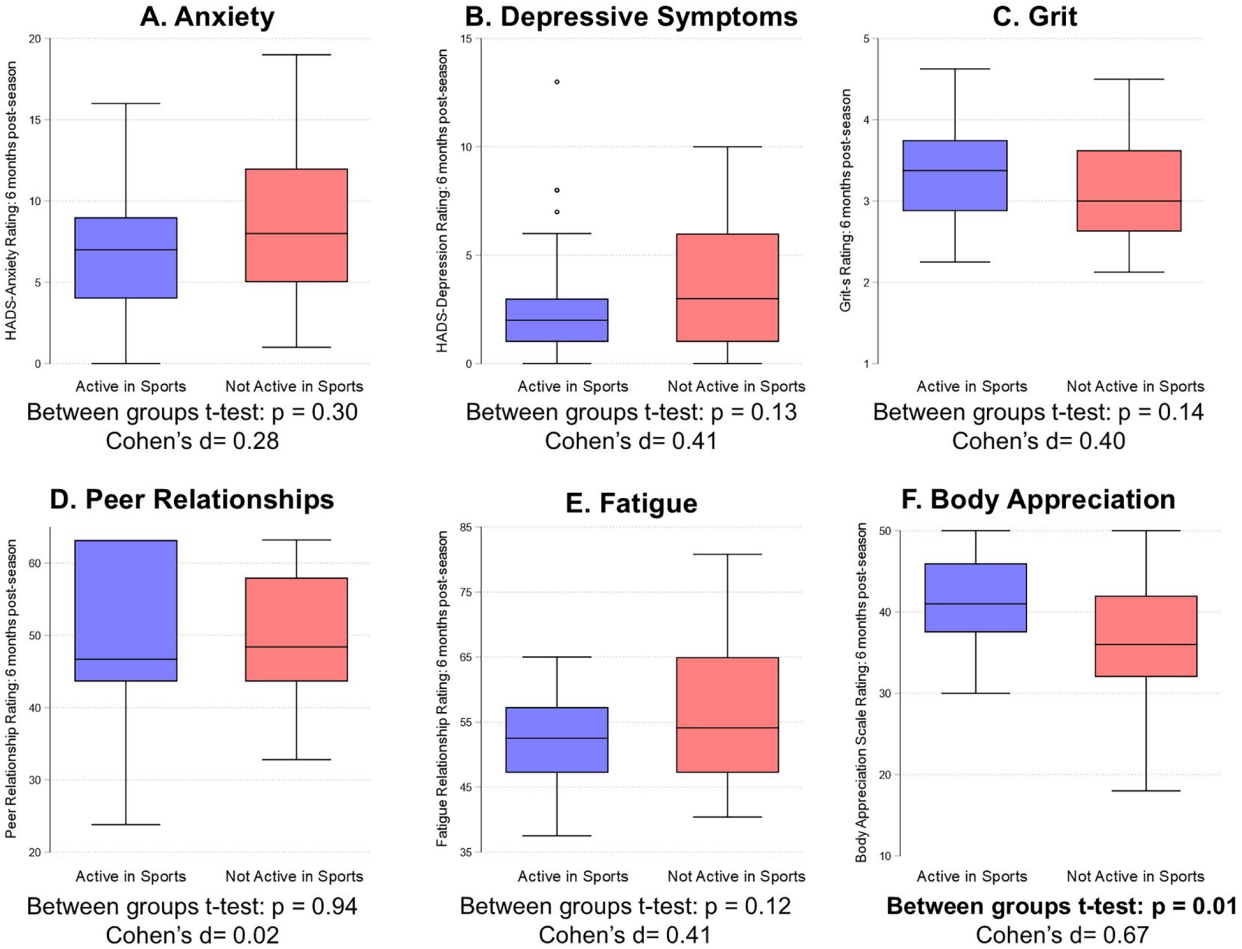
Comparison between adolescent female athletes who did and did not report active organized sport at the 6-month post-season assessment on ratings of **(A)** anxiety, **(B)** depressive symptoms, **(C)** grit, **(D)** peer relationships, **(E)** fatigue, and **(F)** body appreciation.

There were no statistically significant differences between those who did and did not continue to participate in sports at the 6-month follow-up assessment for anxiety ratings, depressive symptoms, grit, peer relationships, or fatigue, with small effect sizes noted for each comparison (Figures 2A–E; Cohen's *d* values = 0.02–0.41). However, those who were actively participating in organized sports at the 6-month follow-up assessment reported significantly higher body appreciation ratings than those who did not, with a moderate effect size noted (Figure 2F; Cohen's *d* = 0.67). After covariate adjustment (post-season rating, physical activity level, age, and BMI), active organized sports participation at the 6-month assessment was significantly associated with better body appreciation ratings (Table 2; *β* = 3.65; 95% CI = 1.08, 6.23; *p* = 0.006), but not with anxiety ratings (*p* = 0.79), depressive symptoms (*p* = 0.46), grit (*p* = 0.08), peer relationships (*p* = 0.39), or fatigue (*p* = 0.33).

**Table 2:** Results from each multivariable regression model evaluating the effect of group (predictor: did and did not continue to participate in sports at the 6-month follow-up assessment) and each outcome measures (anxiety ratings, depressive symptoms, grit, peer relationships, or fatigue, and body appreciation ratings adjusting for covariates (post-season rating, physical activity level, age, and BMI).

Variable	β coefficient	95% Confidence Interval	*P* Value
*Outcome: Anxiety Rating 6 Months After the Season*			
Group (continued sports at 6 months)	−0.26	−2.23, 1.72	0.79
Post-Season Anxiety Rating	0.74	−0.53, 0.95	<0.001
Physical activity level (hours/week)	0.02	−0.11, 0.15	0.80
Age (years)	0.38	−0.47, 1.23	0.37
BMI (kg/m^2^)	−0.09	−0.45, 0.27	0.64
*Outcome: Depressive Symptom Rating 6 months After the Season*			
Group (continued sports at 6 months)	−0.56	−2.08, 0.96	0.46
Post-Season Depressive Symptom Rating	0.55	0.28, 0.81	<0.001
Physical activity level (hours/week)	−0.04	−0.15, 0.06	0.42
Age (years)	0.30	−0.36, 0.97	0.36
BMI (kg/m^2^)	−0.21	−0.49, 0.07	0.14
Grit *Rating 6 Months After the Season*			
Group (continued sports at 6 months)	0.22	−0.02, 0.46	0.08
Post-Season Grit Rating	0.82	0.64, 1.00	<0.001
Physical activity level (hours/week)	0.01	−0.01, 0.02	0.37
Age (years)	0.00	−0.11, 0.11	0.99
BMI (kg/m^2^)	0.01	−0.05, 0.04	0.76
*Peer Relationships Rating 6 Months After the Season*			
Group (continued sports at 6 months)	−1.88	−6.24, 2.47	0.39
Post-Season Peer Relationship Rating	0.80	0.58, 1.01	<0.001
Physical activity level (hours/week)	0.16	−0.13, 0.45	0.27
Age (years)	−0.18	−2.11, 1.75	0.85
BMI (kg/m^2^)	−0.40	−1.20, 0.41	0.33
*Fatigue Rating 6 Months After the Season*			
Group (continued sports at 6 months)	−2.05	−6.21, 2.11	0.33
Post-Season Fatigue Rating	0.57	0.37, 0.76	<0.001
Physical activity level (hours/week)	−0.05	−0.32, 0.23	0.73
Age (years)	−1.13	−2.91, 0.65	0.21
BMI (kg/m^2^)	−0.19	−0.95, 0.56	0.61
*Body Appreciation Rating 6 Months After the Season*			
Group (continued sports at 6 months)	3.65	1.08, 6.23	0.006
Post-Season Body Appreciation Scale Rating	0.76	0.59, 0.93	<0.001
Physical activity level (hours/week)	−0.11	−0.28, 0.06	0.21
Age (years)	−0.18	−1.28, 0.92	0.74
BMI (kg/m^2^)	0.74	0.27, 1.21	0.002

## Discussion

We examined if female adolescent athletes participating in flag football reported different measures of mental health and wellness at an immediate post-season assessment relative to an assessment 6-months following the end of the season. We hypothesized that mental health measures for all 6 domains (anxiety ratings, depressive symptoms, grit, fatigue, peer relationships, and body appreciation) would be different among adolescent females 6 months following the end of the season compared to the immediate post-season assessment. We only observed a significant difference in anxiety ratings, with participants reporting significantly higher anxiety ratings at the 6-month follow-up assessment compared to the post-season assessment. Our hypothesis was otherwise not supported, as there were no significant changes between time points for depressive symptoms, grit, peer relationships, fatigue, or body appreciation.

Our secondary purpose was to examine whether adolescent athletes who were actively participating in organized sports 6 months after the end of the season reported different mental health and wellness ratings compared to athletes who were no longer participating in organized sports or who were out of season. Contrary to our hypothesis, we found no significant differences between those who did and did not report active sports particiatoin at the 6-month follow-up assessment for anxiety ratings, depressive symptoms, grit, peer relationships, or fatigue. However, those who reported active organized sports participation at the 6-month follow-up reported significantly higher body appreciation ratings than those who did not, supporting our hypothesis.

Of the 59 female adolescent athletes in our study, anxiety ratings were worse at 6-month follow-up than immediately post-season. This finding was different than other studies that support long-term sports participation as a method to decrease anxiety levels (8–11). However, in our study sample, only 54% of the participants reported active organized sports participation at the 6-month follow-up and could be one reason for this increased level of anxiety symptoms over time. However, we recognize that this is one of several potential factors that may have influenced this finding, particularly given that anxiety symptoms were rated in reference to the past week, so everyday contextual factors may have influenced ratings. Nonetheless, given the role of routine physical activity as a strategy to cope with anxiety ratings among adolescents, the lack of regular physical activity engagement in about half of our participants may have had some influence within this group change across time. Past work reported that recreational sport athletes with ≥2 years of participation had lower levels of anxiety and depressive symptoms compared to participants that did not participate in recreational sports (8). In contrast, our timeline of a 6-month follow-up assessment may not have been long enough to see pronounced changes across other quality-of-life domains, as the strongest association in this past work was identified for those who participated in organized sports for 4–5 years (8). Lastly, there are multiple variables that could influence anxiety ratings, unrelated to sports participation or physical acitivity, with some examples including stress from school, household environment, and parents or peer relationships. Our findings suggest that female adolescent flag football athletes may require longer than 6 months post-season to see long-term changes for their mental health. Additionally, healthcare providers and coaches should be aware of the changes in anxiety-ratings months after the end of a season, and should promote ongoing sports participation to improve mental health.

We observed that those who reported active organized sports participation at the 6-month follow-up assessment reported higher body appreciation ratings than those who did not. This finding supports the idea that sports participation can serve as a method to enhance body image, particularly among adolescent female athletes. Alternatively, it is possible that individuals with higher body image were more likely to continue participating in sports. Regardless of the causal direction, previous research has consistently demonstrated a strong association between sports participation and improved body image (38–40). A study among Norwegian adolescent athletes observed that sports participation was a positive predictor of body appreciation (38). Other studies have examined the assocations of sport-specific factors and body appreciation among athletes. For example, one study found that there were no significant differences in body appreciation scores among adolescent athletes participating in weight-sensitive sports (i.e., dance, wrestling, gymnastics, running) compared to less weight-sensitive sports (i.e., football, basketball, hockey) (41). Furthermore, adolescent athletes who participated in more years of organized sports and who had better performance results at the most recent competition reported higher body appreciation scores compared to their peers who had fewer years of participation and worse performance results (39). Lastly, in a sample of competitive adolescent rock climbers, body appreciation and competitive success were moderately associated (40). Our sample may have reported higher body appreciation ratings with ongoing sports participation due to the benefit of sports improving self-confidence, self-esteem, and body image. To the best of our knowledge, this study is the first to have assessed longitudinal change in body appreciation related to sports participation among adolescent female athletes. The findings of our study suggest that even 6 months of ongoing sports participation may help promote higher body appreciation scores.

We found no significant differences in depressive symptoms, peer relationships, grit, or fatigue immediately post-season and 6 months later. Contrary to our hypothesis, we did not observe that peer relationship ratings were associated with active organized sports participation. Past work suggests that participants of team sports had greater peer relationships compared to participants of individual sports (20). Team sports, like flag football, encourage social development through teamwork, expanding participants' circle of friends, and building connections. However, our study sample included not only flag football athletes, but those who participated in a variety of other sports as well (both individual and team sports). In addition, persistent physical inactivity in adolescents is associated with poor peer relationships and increased fatigue (21). Our findings may not have shown a significant association between these outcomes and active organized sports participation due to the large percentage (46%) who were not actively involved in playing organized sports at the 6-month follow-up, and the lower physical activity volume of those who were not participating in sports than those who did. Thus, it is important for coaches, parents, and healthcare providers to encourage sustained sports participation for long-term mental health benefits.

Our study had several limitations. We assessed self-reported anxiety and depressive symptom severity through well established and validated questionnaires, and while these are useful ratings-based tools, they were not intended to provide any sort of diagnosis such as what one would receive from a qualified healthcare provider. We also estimated physical activity using one-week self-reported data, which may or may not reflect true physical behavior activity over a longer period of time. In addition, we assessed differences in mental health 6 months post-season to avoid participant dropout, which may not have been a long enough timeframe prospectively to track significant changes. Another limitation is that all participants indicated that they lived in zip codes of the top half of socioeconomic advantage, which limits the generalizability of our findings to those of lower socioeconomic status. Furthermore, we did not include a control group of non-athletes and our sample size was relatively small and included a somewhat homogenous sample of participants. Thus, further study is required to understand how mental health is affected in a group of adolescents who do and do not participate in sports over time. We also acknowledge that only primary sports were identified and reported, and that participants may have participated in additional sports during the study period. However, strengths of our study include the longitudinal study design and examination of mental health measures in a population of adolescent female athletes participating in flag football, which is a newer and less-studied sport.

## Conclusion

Adolescent female athletes participating in flag football reported significantly higher anxiety ratings at the 6-month follow-up assessment compared to the immediate post-season assessment, and nearly half reported not participating in organized sports at the 6-month follow-up assessment. There were no significant differences across time for depressive symptoms, grit, peer relationships, fatigue, or body appreciation. However, those who reported active organized sports particaiptoin at the 6-month follow-up assessment reported significantly higher body appreciation ratings than those who did not. Our findings suggest that healthcare providers, coaches, and parents should encourage long-term participation in sports for improved body appreciation scores among female adolescent athletes. Additionally, as anxiety ratings increased 6 months following the end of a sports season, participating in multiple seasons may help improve long-term mental health among adolescent athletes. As flag football increases in popularity and is an additional team sport opportunity among adolescent female athletes, future directions include prospectively examining the relationship of mental health measures and engagement in organized sports into adulthood. It is important in future work to monitor mental health measures at various time points post-season to better understand how sustained sports participation affects mental health measures long-term.

## Data Availability

The raw data supporting the conclusions of this article will be made available by the authors, upon reasonable request.
